# Transcriptome Sequencing Reveals *Salmonella* Flagellin Activation of Interferon-β-Related Immune Responses in Macrophages

**DOI:** 10.3390/cimb45040183

**Published:** 2023-03-27

**Authors:** Li Song, Dan Xiong, Yaya Wen, Ruimeng Tan, Xilong Kang, Xinan Jiao, Zhiming Pan

**Affiliations:** 1 Jiangsu Co-Innovation Center for Prevention and Control of Important Animal Infectious Diseases and Zoonoses, Yangzhou University, Yangzhou 225009, China; 2 Jiangsu Key Laboratory of Zoonosis, Yangzhou University, Yangzhou 225009, China; 3 Key Laboratory of Prevention and Control of Biological Hazard Factors (Animal Origin) for Agrifood Safety and Quality, Ministry of Agriculture and Rural Affairs, Yangzhou University, Yangzhou 225009, China

**Keywords:** *Salmonella* flagellin, macrophage, RNA-Seq, immune response, interferon-β, ISGs

## Abstract

The flagellin (FliC) of *Salmonella typhimurium* is a potential vaccine adjuvant as it can activate innate immunity and promote acquired immune responses. Macrophages are an important component of the innate immune system. The mechanism of flagellin’s adjuvant activity has been shown to be related to its ability to activate macrophages. However, few studies have comprehensively investigated the effects of *Salmonella* flagellin in macrophages using transcriptome sequencing. In this study, RNA-Seq was used to analyze the expression patterns of RAW264.7 macrophages induced by FliC to identify novel transcriptomic signatures in macrophages. A total of 2204 differentially expressed genes were found in the FliC-treated group compared with the control. Gene ontology and KEGG pathway analyses identified the top significantly regulated functional classification and canonical pathways, which were mainly related to immune responses and regulation. Inflammatory cytokines (IL-6, IL-1β, TNF-α, etc.) and chemokines (CXCL2, CXCL10, CCL2, etc.) were highly expressed in RAW264.7 cells following stimulation. Notably, flagellin significantly increased the expression of interferon (IFN)-β. In addition, previously unidentified IFN regulatory factors (IRFs) and IFN-stimulated genes (ISGs) were also significantly upregulated. The results of RNA-Seq were verified, and furthermore, we demonstrated that flagellin increased the expression of IFN-β and IFN-related genes (IRFs and ISGs) in bone marrow-derived dendritic cells and macrophages. These results suggested that *Salmonella* flagellin can activate IFN-β-related immune responses in macrophages, which provides new insight into the immune mechanisms of flagellin adjuvant.

## 1. Introduction

In recent years, there have been various outbreaks of infectious diseases in livestock and poultry, such as avian influenza and African swine fever [1,2], which have caused serious economic losses in these industries. Human infections resulting from pathogens derived from contaminated meat or the environment, and the consequential impacts on human health, cause widespread concern [2]. Vaccinations are an important measure to prevent infectious diseases, and adjuvants play an important role in enhancing the immune response induced by vaccines. The research and development of vaccine adjuvants is, therefore, of great significance.

Flagellin, the main structural protein of bacterial flagella, is a vaccine adjuvant with a wide range of potential applications [3]. As a pathogen-associated molecular pattern (PAMP), flagellin can be recognized by host cell pattern recognition receptors (PRRs), membrane-bound Toll-like receptor (TLR) 5, and the cytosolic sensor NOD-like receptor (NLR) family CARD domain-containing protein (NLRC) 4, to induce a variety of cytokines and chemokines, effectively activating innate immunity and promoting acquired immune responses [4,5,6]. Flagellin has strong plasticity and can be combined with exogenous antigens in a variety of ways. Previously, we applied *Salmonella typhimurium* flagellin (FliC) to an influenza subunit vaccine, and demonstrated that FliC could significantly promote immune responses in both mouse and poultry models, with good adjuvant activity [7,8]. 

In recent decades, the mechanism of action of flagellin adjuvant has been studied and the PAMPs–PRRs recognition model was originally proposed [9]. Flagellin can promote the activation and maturation of antigen presenting cells (APCs) and secrete inflammatory cytokines, chemokines, and costimulators [10]. Activated APCs, in turn, activate an antigen-specific acquired immune response [11]. Activation of the TLR5 signaling pathway is important for flagellin to exert its adjuvant effect, since most flagellin-based vaccines activate APCs outside of the target cell. However, previous studies in our laboratory have shown that flagellin retains high adjuvant activity in TLR5-deficient mice [12], and other studies have shown that the flagellin-induced antibody response is independent of the TLR5 and NLRC4 inflammasome pathways [13]. Therefore, we suppose that flagellin may also be involved in the transduction of other intracellular signaling pathways, and the molecular mechanism by which it activates innate immunity deserves further study. 

Transcriptome sequencing (RNA-Seq) technology is increasingly used to study gene expression, as this method can identify novel transcripts, and can be extremely accurate when sufficient coverage is obtained. Furthermore, validation techniques, such as quantitative real-time PCR (qRT-PCR), have corroborated the accuracy of RNA-Seq [14]. However, few studies have applied this approach to analyze the effects of flagellin. Therefore, in this study, FliC was used to stimulate the murine macrophage RAW264.7 cell line, and a comprehensive transcriptional profile of responses to flagellin was obtained using RNA-Seq to gain new insight into the molecular mechanisms of the action of flagellin. We found that *Salmonella* FliC significantly increased the expression of IFN-β and IFN-stimulated genes (ISGs) in murine macrophages in vitro, and promoted IFN-β-related immune responses in bone marrow-derived macrophages (BMMs) and dendritic cells (BMDCs) ex vivo. 

## 2. Materials and Methods

### 2.1. Mice and Ethics Statement

Female, 6–8-week-old C57BL/6 mice were purchased from Beijing Vital River Laboratory Animal Technology Co. Ltd. All mice were housed in isolators with controlled temperature, light, and ventilation. Pathogen-free water and food were supplied ad libitum. All animal studies were approved by the Committee on the Ethics of Animal Experiments of Yangzhou University (Approval ID: SYXK (Su) 2017-0044). 

### 2.2. Extraction and Characterization of Flagellin (FliC) 

*Salmonella typhimurium* strain ATCC14028s (pTrc99a-fliC-WT) was cultured in the presence of 100 mg/mL ampicillin, and the isolation of highly purified flagellin (FliC) was performed according to a well-established method [15]. The extracted FliC protein was analyzed by sodium dodecyl sulfate polyacrylamide gel electrophoresis (SDS-PAGE) and western blotting with a mouse polyclonal antibody specific for FliC (anti-FliC). Endotoxin was removed from the protein using the ProteoSpin™ Endotoxin Removal Kit Maxi for protein and peptides (Norgen Biotek Corp., Thorold, ON, Canada). Residual endotoxin levels were measured using a Chromogenic Endpoint Tachypleus Amebocyte Lysate (CE TAL) Assay Kit (Chinese Horseshoe Crab Reagent Manufactory Co., Xiamen, China) until the final endotoxin content was less than 0.05 EU/μg.

### 2.3. TLR5 Ligand Activity of Flagellin

HEK293-mTLR5 cells were cultured in a 96-well microtiter plate at a seeding density of 5 × 10^4^ in complete Dulbecco’s modified Eagle’s medium (DMEM) containing 10% fetal bovine serum (FBS) and 1% penicillin-streptomycin (Gibco, Carlsbad, CA, USA) overnight. The next day, cells were treated with 100 ng/mL of the FliC protein. After 5 h of stimulation, the cell supernatants were harvested, and IL-8 expression was evaluated by an enzyme-linked immunosorbent assay (ELISA) using the Human IL-8 Ready-SET-Go ELISA Set (eBioscience, San Diego, CA, USA).

### 2.4. Flagellin Stimulation of RAW264.7 Cells at Different Concentrations

The murine macrophage cell line RAW264.7 was cultured in a 24-well plate at a seeding density of 2 × 10^5^ in complete DMEM containing 10% FBS and 1% penicillin-streptomycin overnight. The next day, cells were treated with 0.01, 0.1, 1, or 5 μg/mL endotoxin-free FliC. After 5 h of stimulation, the cells were collected for RNA extraction. The mRNA levels of cytokines (IL-1β and IL-6) and chemokines (CXCL2 and CXCL10) were measured using qRT-PCR to evaluate the optimal stimulating concentration of flagellin to activate RAW264.7 cells.

### 2.5. RNA-Seq and Data Analysis 

The most suitable flagellin concentration to activate RAW264.7 cells was chosen for subsequent RNA-Seq experiments given the robust response at this dose. The total RNA from FliC-stimulated and unstimulated RAW264.7 cells (three samples for each group) was sent to Novogene Co., Ltd. for RNA-Seq (Beijing, China, http://www.novogene.com/, accessed on 11 September 2017). After quality testing, cDNA libraries were constructed and the samples were sequenced using the Illumina HiSeq PE150 platform. The sequence reads were mapped to the mouse reference genome (GCA_001632525.1) and normalized to fragments per kilobase of transcript per million mapped reads (FPKM) values. For RNA-Seq analysis, HTseq (v0.6.1) software was used to analyze the gene expression level of each sample, and the model used was UNION. DESeq (1.10.1) software was used for differential expression analysis (Padj < 0.05). The R package pheatmap (v1.0.12) software (log2 fold change ≥ 1.0) was used for cluster analysis of differential genes. The GOseq (Release 2.12) and KOBAS (v2.0) software packages were used for gene ontology (GO) and KEGG metabolic pathway enrichment analysis (corrected_*p*-value < 0.05). 

### 2.6. Verification of RNA-Seq

According to the results of RNA-Seq analysis, some significantly upregulated immune-related differentially expressed genes (DEGs) were selected for verification. The RAW264.7 cells were treated with 1 μg/mL endotoxin-free FliC. After 5 h of stimulation, the cells were collected for RNA extraction. The mRNA levels of the DEGs were measured using qRT-PCR. To further verify that flagellin itself had the ability to induce IFN-β, RAW264.7 cells were stimulated by FliC (1 μg/mL), FliC + Trypsin, LPS (0.1 μg/mL) and LPS + Trypsin groups. Trypsin pre-treatment was conducted by incubation of FliC or LPS with Trypsin (1:2 *v*/*v*) at 37 °C for 10 min before stimulation. DMEM and Trypsin groups were set as negative controls. After 5 h of stimulation, cells and cultural supernatants were collected, respectively. The mRNA level of IFN-β was detected by qRT-PCR, and the secretion level of IFN-β in the supernatant was detected by IFN-β DuoSet ELISA kit (R&D Systems).

### 2.7. Flagellin Stimulation of TLR4-Deficient HCT116-Dual Cells

The human colorectal carcinoma cell line HCT116-Dual (InvivoGen, Cat# hctd-nfis) was used to verify the induction of IFN-β by flagellin independent of LPS/TLR4. HCT116-Dual cells express numerous PRRs but not TLR4. HCT116-Dual cells allow for the simultaneous study of the nuclear transcription factor κB (NF-κB) pathway, by assessing the activity of secreted embryonic alkaline phosphatase (SEAP), and the interferon regulatory factor (IRF) pathway, by monitoring the activity of Lucia luciferase. HCT116-Dual cells were cultured in a 96-well plate at a seeding density of 5 × 10^4^ in complete DMEM containing 10% FBS, 10 µg/mL blasticidin, and 100 µg/mL Zeocin overnight. The next day, cells were treated with endotoxin-free FliC (1 μg/mL), poly(I:C) as positive control (2.5 μg/mL), and LPS as negative control (0.1 μg/mL). After 5 h of stimulation, the induction of IRF and NF-κB reporter proteins was measured in the cell culture supernatant by using QUANTI-Blue, a SEAP detection reagent, and QUANTI-Luc, a Lucia detection reagent (InvivoGen) with a microplate reader (BioTek, Winooski, VT, USA).

### 2.8. Expression Levels of IFN-β in RAW264.7 Cells Stimulated by Polymyxin B (PMB)-Treated FliC

To further eliminate the influence of LPS contamination, polymyxin B was used to treat the FliC protein. RAW264.7 macrophages were cultured in a 24-well plate at a seeding density of 2 × 10^5^ in complete DMEM containing 10% FBS and 1% penicillin-streptomycin overnight. The next day, cells were treated with endotoxin-free FliC (1 μg/mL), FliC + PMB, LPS (0.1 μg/mL) and LPS + PMB. PMB pre-treatment was conducted by the incubation of FliC or LPS with PMB (10 μg/mL) at 37 °C for 2 h before stimulation. DMEM and PMB groups were set as negative controls. After 5 h of stimulation, the cells were collected for RNA extraction. The mRNA levels of IFN-β, IL-1β, and IL-6 were measured using qRT-PCR.

### 2.9. Flagellin Stimulation of Human THP-1 Cells

The human THP-1 cells were seeded in complete RPMI 1640 (Gibco) medium at a density of 2 × 10^5^ cells/well in 24-well plates. The next day, cells were stimulated with FliC (1 µg/mL) or LPS (0.1 µg/mL), and the unstimulated group was included as a negative control. After 5 h of stimulation, the cells were collected for RNA extraction, and the mRNA levels of IFN-β, IL-1β, and IL-8 were determined by qRT-PCR.

### 2.10. Isolation of Bone Marrow-Derived Macrophages (BMMs) and Dendritic Cells (BMDCs)

BMMs and BMDCs were generated from bone marrow as previously described [16]. Briefly, 6-week-old C57BL/6 mice were euthanized, and the bone marrow cells from femurs and tibias were cultured in complete RPMI 1640 medium containing 10% FBS and 1% penicillin-streptomycin. For the induction of BMDCs, 10 ng/mL granulocyte-macrophage colony-stimulating factor (GM-CSF) and IL-4 (R&D Systems, Minneapolis, MN, USA) were supplemented, and for the induction of BMMs, 10 ng/mL M-CSF (R&D Systems) was added to the complete medium. The cultured cells were incubated in 5% CO_2_ at 37 °C for 7 days, and one third of the total media volume was replaced with fresh complete medium at days 3 and 5. On day 7 of culture, BMMs and BMDCs were harvested by scraping the cells into ice-cold PBS.

### 2.11. Flagellin Stimulation of BMMs and BMDCs

BMMs and BMDCs were seeded in complete RPMI 1640 medium at a density of 4 × 10^5^ cells/well in 24-well plates. For some experiments, 8 × 10^4^ cells/well were cultured in 96-well plates. The next day, cells were stimulated with FliC (1 µg/mL) or lipopolysaccharide (LPS) (0.1 µg/mL), and the unstimulated group was included as a negative control. After 5 h, the cells in the 24-well plate were collected for RNA extraction, and the mRNA levels of IL-1β, IL-6, IFN-β, and IFN-related genes (IRFs and ISGs) were determined by qRT-PCR. The cells in the 96-well plate were treated for 12 h. After treatment, IFN-β secretion in the cell supernatants was measured using an IFN-β DuoSet ELISA kit.

### 2.12. RNA Extraction, Reverse Transcription, and qRT-PCR

Total RNAs from RAW264.7, THP-1, BMMs, and BMDCs were extracted using the RNeasy Plus Mini Kit (Qiagen, Mannheim, Germany) and reverse transcribed into cDNA with the PrimeScript RT Reagent Kit (Takara, Dalian, China), followed by qRT-PCR analysis using SYBR Green master mix (Roche Diagnostics, Tokyo, Japan) on the ABI 7500 Fast Real-Time PCR System. PCR amplification was performed in a total volume of 20 μL containing 10 μL of 2 × SYBR Premix Ex Taq II, 2 μL of the diluted cDNA, and 0.8 μL of each primer. The real-time PCR program started with denaturing at 95 °C for 30 s, followed by 40 cycles of 95 °C for 5 s and 60 °C for 60 s. Relative quantifications of the mRNA of genes are shown as the comparative threshold cycle number for each sample (2^−^*^ΔΔCT^*). Gene expression was compared with the corresponding GAPDH level. The primers used for qRT-PCR were synthesized by GenScript Biotechnology Co., Ltd. (Nanjing, China), and the sequences are shown in Table 1.

### 2.13. ELISA to Detect the Secretion Level of IFN-β

The secretion levels of IFN-β in the cell supernatants of mouse BMMs and BMDCs were measured using the IFN-β DuoSet ELISA kit (R&D Systems) according to the manufacturer’s instructions. Briefly, the captured antibodies were diluted with PBS to a working concentration, added at 100 μL/well to a 96-well plate, and incubated overnight at room temperature. The next day, 100 μL/well of the diluted cell supernatant sample or standard was added and incubated at room temperature for 2 h. Then, 100 μL/well of antibody diluent was added for 2 h, followed by 100 μL/well of streptavidin-HRP diluent for 20 min. Finally, 100 μL/well of substrate solution was added and incubated at room temperature for 20 min, followed by 50 μL/well of stop solution. The OD_450_ and OD_570_ values were determined using a microplate reader (BioTek, Winooski, VT, USA).

### 2.14. Statistical Analysis

All data are expressed as the mean ± standard error of the mean (SEM). The statistical analysis was performed using GraphPad 5.0 software, and the data were analyzed using the Student’s *t* test and one-way ANOVA. *p* < 0.05 (*), *p* < 0.01 (**), and *p* < 0.001 (***) were considered significant.

## 3. Results

### 3.1. Identification of the Flagellin from Salmonella typhimurium

**Figure 1:**
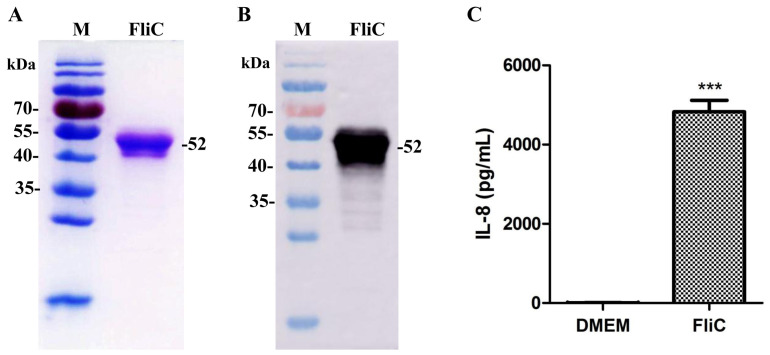
Identification of the flagellin from *Salmonella typhimurium*. (**A**) The extracted flagellin (FliC) of *S. typhimurium* ATCC14028s (pTrc99a-fliC-WT) was analyzed by SDS-PAGE, and (**B**) the mouse anti-FliC polyclonal antibody was used for western blot analysis. (**C**) HEK293-mTLR5 cells were stimulated with FliC, and the IL-8 secretion level in the cell supernatant was detected for TLR5 ligand activity. *** *p* < 0.001 was considered significant.

The extracted flagellin (FliC) of *S. typhimurium* ATCC14028s (pTrc99a-fliC-WT) was characterized by SDS-PAGE and western blotting. The FliC protein was successfully extracted with a clear band of 52 kDa (Figure 1A). As expected, FliC reacted strongly with the anti-FliC polyclonal antibody, indicating that the extracted flagellin had good immune reactivity (Figure 1B). In an IL-8 secretion assay, HEK293-mTLR5 cells were stimulated with endotoxin-free FliC. Compared with the control group, the IL-8 secretion level in the cell supernatant was significantly increased in the FliC-treated group (Figure 1C). These results showed that the extracted flagellin had good biological activity.

### 3.2. The Optimal Concentration of Flagellin to Activate RAW264.7 Cells

**Figure 2:**
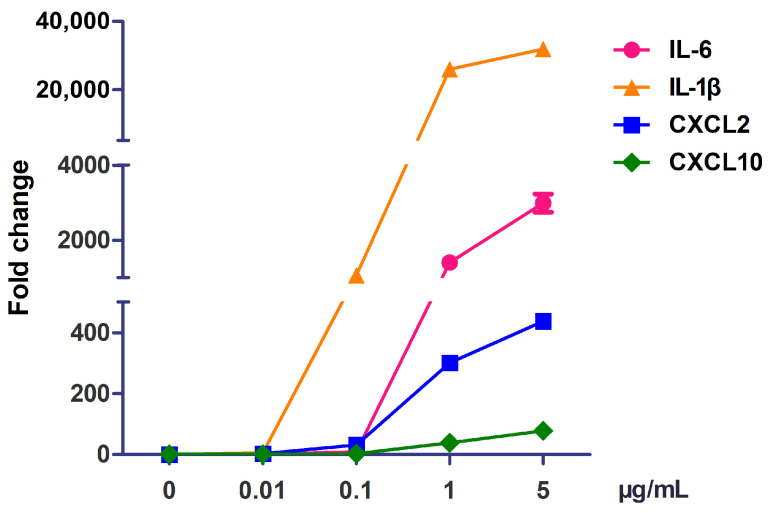
Dose response characteristic of RAW264.7 cells to flagellin. The mRNA levels of cytokines and chemokines (IL-1β, IL-6, CXCL2, and CXCL10) related to inflammatory responses were measured after 5 h of stimulation with flagellin at 0.01, 0.1, 1, and 5 μg/mL using qRT-PCR. A flagellin concentration of 1 μg/mL was chosen for subsequent experiments given the robust response at this dose.

In RNA-Seq studies, the preparation of biological samples is a significant initial step. To determine the appropriate dose response, we performed expression analysis in RAW264.7 cells treated with different concentrations (0.01, 0.1, 1, and 5 μg/mL) of FliC. We observed the dose-dependent upregulation of cytokines (IL-1β and IL-6) and chemokines (CXCL2 and CXCL10) after FliC treatment. Most of these genes were upregulated significantly at the 1 and 5 μg/mL dose points, and there was no significant difference between the two groups (Figure 2). Therefore, RAW264.7 cells stimulated with 1 μg/mL FliC were chosen for subsequent RNA-Seq experiments given the robust response at this dose. 

### 3.3. RNA-Seq Transcriptional Profiling in FliC-Stimulated RAW264.7 Cells

**Figure 3:**
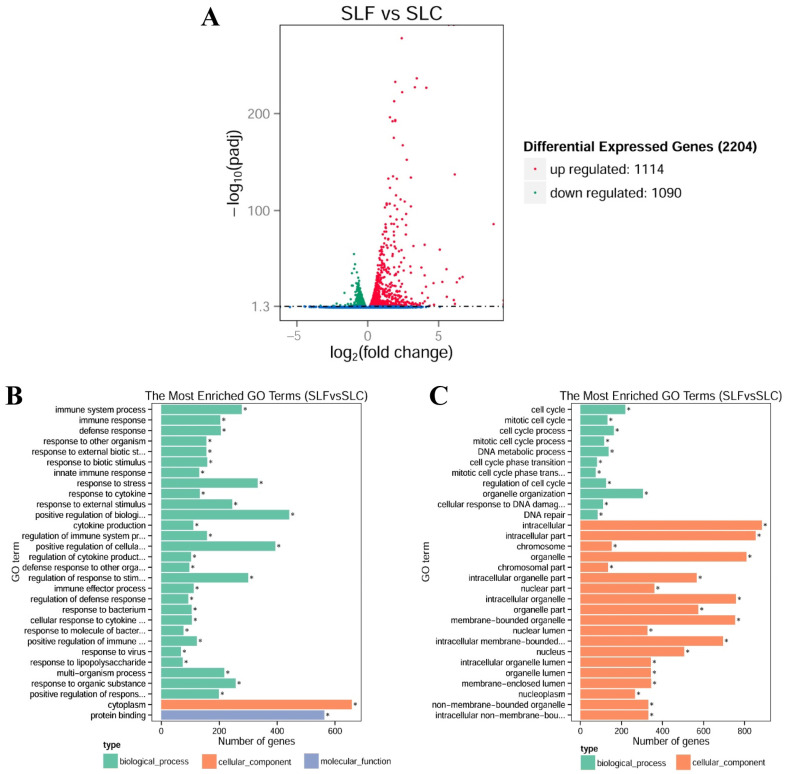
Volcano plot and gene ontology (GO) enrichment analysis of DEGs. RNA-Seq analysis of the differentially expressed genes (DEGs) at 5 h after FliC stimulation in the RAW264.7 cells. (**A**) Volcano plot of DEGs. The *x*-axis indicates the difference in expression level on a log2-fold change; the *y*-axis represents statistically significant differences in gene expression on a negative log10 (adjusted *p*-values). Red indicates upregulated genes and green indicates downregulated genes. (**B**) Top 30 GO terms based on upregulated DEGs, and (**C**) top 30 GO terms based on downregulated DEGs. The *x*-axis is the number of DEGs in this term; the *y*-axis is the enrichment GO term. Different colors are used to distinguish biological processes, cellular components, and molecular functions. SLC represents the DMEM control group, and SLF represents the FliC-stimulated group. * indicates a significantly enriched GO term.

**Figure 4:**
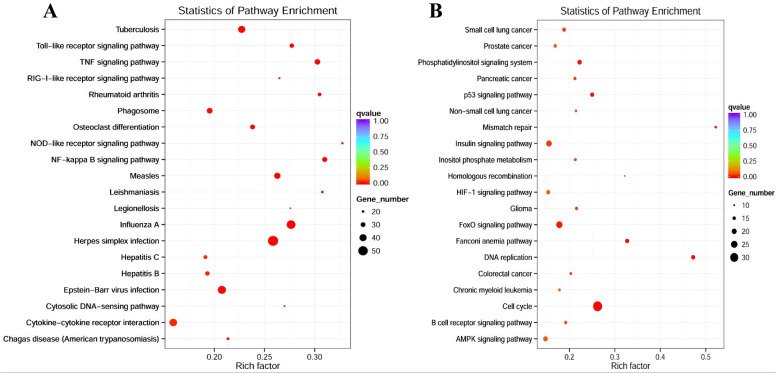
KEGG enrichment analysis. The top 20 upregulated (**A**) and downregulated (**B**) KEGG pathway items in FliC-stimulated RAW264.7 cells compared with control cells are presented. Circles present the gene number. The color of the circles indicates the q-value. Significant pathway enrichment indicates the main biochemical pathways and signal transduction pathways that the DEGs are involved in. All of the genes were used for KEGG ontology (KO) enrichment analyses.

To gain a comprehensive understanding of the transcriptomic profile of RAW264.7 cells after flagellin stimulation, RNA-Seq experiments were performed. The RNA-Seq analysis revealed a total of 2204 DEGs when comparing the FliC-treated group with the control group (Padj < 0.05), of which 1114 genes were upregulated and 1090 genes were downregulated (Figure 3A). This indicated that flagellin has a significant effect on the expression level of the macrophage transcriptome. For reference, the top upregulated genes (log2-fold ≥ 2.0) and downregulated genes (log2-fold ≤ −1.0) upon *Salmonella* FliC stimulation are presented in Supplementary Table S1 and Table S2, respectively. The results showed that most of the upregulated genes had a larger fold difference than the downregulated genes. According to GO enrichment analysis, DEGs were divided into three groups: those involved in biological processes, those that are cellular components, and those with molecular functions. We observed that the top 30 GO terms based on upregulated DEGs were mainly involved in immune responses and regulation processes (Figure 3B). The top 30 GO terms based on downregulated DEGs were mainly related to cell cycle and cellular components (Figure 3C). To better understand the molecular functions of DEGs, we analyzed the KEGG enrichment pathway. According to the top 20 upregulated pathway items that showed the most significant enrichment (Figure 4A), several well-known signaling pathways that are important in immune responses were activated by flagellin, such as the TLR signaling pathway, the tumor necrosis factor (TNF) signaling pathway, the RIG-I-like receptor signaling pathway, the NLR signaling pathway, and the NF-κB signaling pathway. Among them, the RIG-I-like signaling pathway has not previously been reported to be activated by flagellin. Following analysis of the top 20 downregulated pathway items that showed the most significant enrichment (Figure 4B), the main signaling pathways were associated with cancer and cell cycles. These findings were interesting and confirmed that the experimental approach was effective.

**Figure 5:**
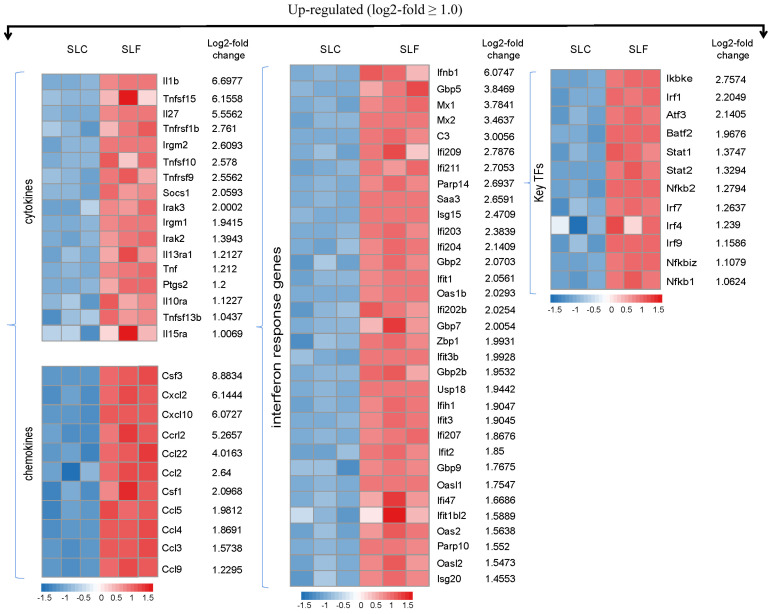
Cluster analysis of DEGs. The heatmap representing RNA-Seq gene expression data for upregulated (log2-fold change ≥ 1.0) immune-related genes after 5 h of FliC stimulation in RAW264.7 cells compared with control cells. The heatmap was generated with the R package pheatmap (v1.0.12) software. SLC represents the DMEM control group, and SLF represents the FliC-stimulated group.

As most of the downregulated genes were not associated with immune responses, only the upregulated DEGs were studied further. To aid the visualization of the upregulated DEG expression pattern involved in the immune-related signaling pathway, heat maps were generated with the R package pheatmap (v1.0.12) software (log2 fold change ≥ 1.0). RNA-Seq analysis revealed that most of the proinflammatory cytokines and chemokines were highly induced in RAW264.7 cells. These included interleukin-related genes (IL-1β, IL-6, and IL-18), TNF, C-C motif chemokine ligand 2, 3, 4, and 5 (CCL2, CCL3, CCL4, and CCL5, respectively), C-X-C motif chemokine ligand 2 and 10 (CXCL2 and CXCL10, respectively). The activation of TLR signaling pathways led to the induced transcription of various immune-related genes. Importantly, we observed that type I interferon (IFN-β) and ISGs were also significantly upregulated in expression in FliC-treated RAW264.7 cells. These highly induced genes included the myxovirus resistance genes (Mx1 and Mx2), the IFN-induced protein with the tetratricopeptide (IFIT) family of genes (IFIT1, IFIT2, and IFIT3b), and the IRF3-dependent genes (ISG15, ISG20, oligoadenylate synthetase 1B (OAS1b), etc.) (Figure 5).

### 3.4. Verification of DEGs Identified by RNA-Seq by qRT-PCR

**Figure 6:**
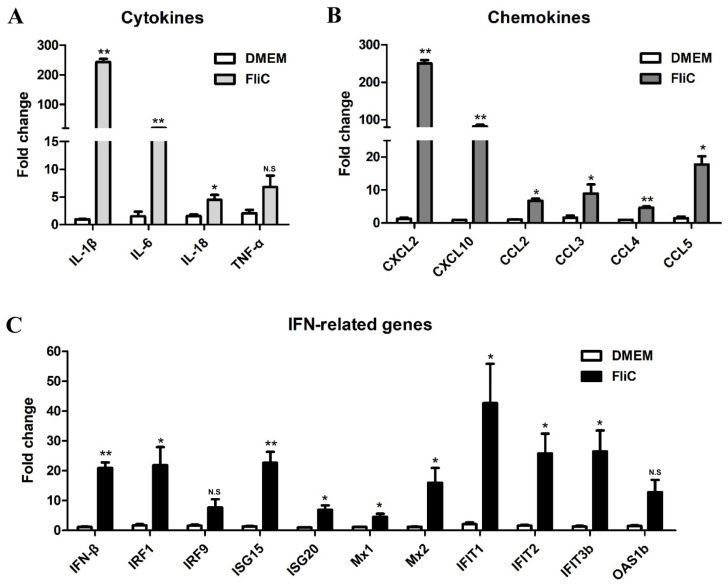
Verification of upregulated genes in RAW264.7 cells. Some of the upregulated immune-related genes in the KEGG signaling pathways were selected for qRT-PCR verification. The mRNA levels of inflammatory cytokines (**A**), chemokines (**B**), and interferon-related genes (**C**) related to immune responses were measured after 5 h of stimulation with flagellin at 1 μg/mL using qRT-PCR. * *p* < 0.05 and ** *p* < 0.01 compared with the control. N.S., not significant.

A large number of the genes that were identified to be differentially regulated using RNA-Seq analysis were subjected to validation using qRT-PCR. For inflammatory-related cytokines and chemokines, and 13 upregulated DEGs were selected for verification, and the RNA-Seq expression patterns were confirmed for 10 of these genes (IL-1β, IL-6, IL-18, TNF-α, CXCL2, CXCL10, CCL2, CCL3, CCL4, and CCL5) (Figure 6A,B). Two genes (IL-27 and CCL22) were not significantly altered in qRT-PCR analysis compared with the RNA-Seq experiments. Furthermore, 11 of the upregulated DEGs enriched in IFN-related signaling pathways were selected for validation by qRT-PCR: IFN-β, its upstream genes (IRF1 and IRF9), and its downstream ISGs (ISG15, ISG20, Mx1, Mx2, IFIT1, IFIT2, IFIT3b, and OAS1b). The mRNA levels of these genes were upregulated in RAW264.7 cells induced by flagellin compared with unstimulated cells, which further confirmed the RNA-Seq results. Among them, IFN-β, IRF1, ISG15, Mx2, IFIT1, IFIT2, and IFIT3b were most significantly upregulated in the qRT-PCR analysis (Figure 6C).

### 3.5. Expression and Secretion Levels of IFN-β in RAW264.7 Cells Stimulated by Trypsin-Treated FliC

**Figure 7:**
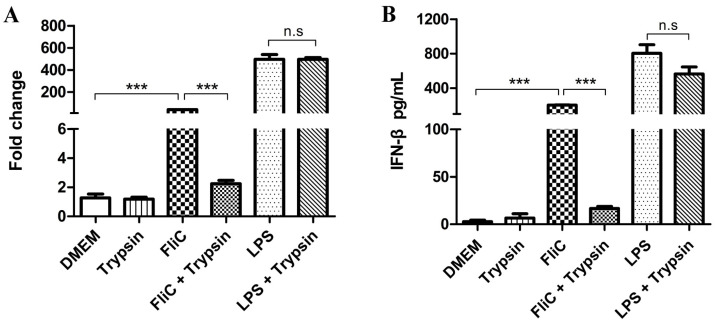
Expression and secretion levels of IFN-β in RAW264.7 cells stimulated by Trypsin-treated FliC. RAW264.7 cells were treated with FliC, FliC + Trypsin, LPS, or LPS + Trypsin groups. Trypsin pre-treatment was conducted by incubation of FliC or LPS with Trypsin (1:2 *v*/*v*) at 37 °C for 10 min before stimulation. DMEM and Trypsin groups were set as negative controls. After 5 h of stimulation, cells and cultural supernatants were collected. (**A**) The mRNA levels of IFN-β were determined by qRT-PCR, and (**B**) the secretion levels of IFN-β in culture medium were measured using an ELISA set. *** *p* < 0.001 was considered significant. n.s., not significant.

To further prove that flagellin itself could induce the up-regulated expression of IFN-β in RAW264.7, Trypsin-treated FliC was used to stimulate cells to detect the expression and secretion level of IFN-β. The results showed that when compared with the DMEM and Trypsin control groups, the FliC could significantly upregulate the expression of IFN-β, and there was no significant difference between the FliC + Trypsin group and the control group. However, compared with the FliC group, the mRNA level of IFN-β in the FliC + Trypsin group was significantly decreased, indicating that FliC could not promote the upregulated expression of IFN-β after the Trypsin treatment. Meanwhile, it was observed that both the LPS and LPS + Trypsin groups could significantly induce the upregulation of IFN-β after the stimulation of the cells, and there was no significant difference between the two groups (Figure 7A). In addition, the same results were also observed at the protein levels (Figure 7B). These results showed that Trypsin could digest FliC protein, but could not digest LPS, and the Trypsin treatment did not affect the effect of LPS in RAW264.7, which further proved that flagellin could induce the upregulated expression of IFN-β in RAW264.7 macrophages.

### 3.6. Activation of IRF Pathway in TLR4-Deficient HCT116-Dual Cells Stimulated by FliC

**Figure 8:**
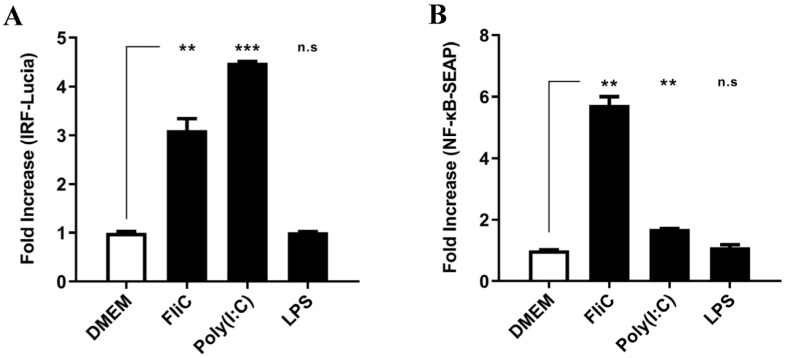
Activation of IRF pathway in TLR4-deficient HCT116-Dual cells stimulated by FliC. The human colorectal carcinoma cell line HCT116-Dual cells were cultured in a 96-well plate at a seeding density of 5 × 10^4^ in complete DMEM containing 10% FBS, 10 µg/mL blasticidin, and 100 µg/mL Zeocin overnight. The next day, cells were treated with endotoxin-free FliC (1 μg/mL), poly(I:C) as the positive control (2.5 μg/mL), and LPS as the negative control (0.1 μg/mL). After 5 h of stimulation, the induction of IRF and NF-κB reporter proteins were measured in the cell culture supernatant by using QUANTI-Luc, a Lucia detection reagent (**A**), and QUANTI-Blue, a SEAP detection reagent (**B**) with a microplate reader. ** *p* < 0.01 and *** *p* < 0.001 compared with the control. n.s., not significant.

To verify the induction of IFN-β by flagellin was independent of LPS/TLR4, the TLR4-deficient HCT116-Dual cells were used. HCT116-Dual cells allow to simultaneously study the NF-κB pathway, by assessing the activity of SEAP, and the IRF pathway, by monitoring the activity of Lucia luciferase. HCT116-Dual cells were stimulated with FliC, poly(I:C) as positive control or LPS as negative control. The results showed that FliC induced the activation of both the IRF and NF-κB pathways significantly. However, LPS stimulation had no effects on either pathway in the TLR4-deficient cells (Figure 8). These results indicate that flagellin could activate the IFN-β signaling independent of LPS/TLR4.

### 3.7. Induction of IFN-β in RAW264.7 Cells Stimulated by Polymyxin B-Treated FliC

**Figure 9:**
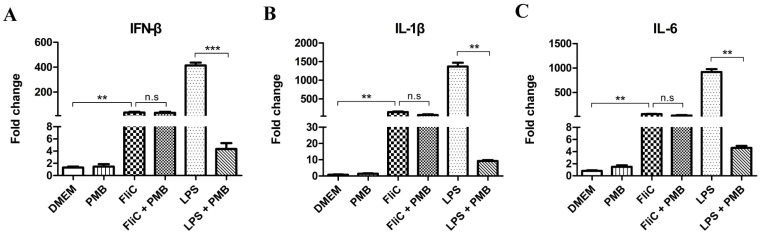
Expression levels of IFN-β, IL-1β, and IL-6 in RAW264.7 cells stimulated by polymyxin B-treated FliC. RAW264.7 cells were treated with endotoxin-free FliC (1 μg/mL), FliC + PMB, LPS (0.1 μg/mL), and LPS + PMB. PMB pre-treatment was conducted by incubation of FliC or LPS with PMB (10 μg/mL) at 37 °C for 2 h before stimulation. DMEM and PMB groups were set as negative controls. After 5 h of stimulation, the mRNA levels of IFN-β (**A**), IL-1β (**B**), and IL-6 (**C**) were determined by qRT-PCR. ** *p* < 0.01 and *** *p* < 0.001 were considered significant. n.s., not significant.

To further eliminate the influence of LPS, PMB-treated FliC was used to stimulate RAW264.7 cells to detect the expression level of IFN-β, IL-1β, and IL-6. The results showed that compared with the DMEM control group, FliC could significantly upregulate the expression of IFN-β, IL-1β, and IL-6, and there was no significant difference between the FliC group and the FliC + PMB group. Meanwhile, it was observed that LPS group could induce significantly upregulation of the cytokines after the stimulation of the cells. However, compared with the LPS group, the induction levels of IFN-β, IL-1β, and IL-6 in the LPS + PMB group were significantly decreased, indicating that LPS was digested successfully after the PMB treatment (Figure 9). These results showed that the PMB treatment did not affect the effect of FliC in RAW264.7, which further proved that flagellin could induce the upregulated expression of IFN-β in RAW264.7 macrophages.

### 3.8. Flagellin Stimulates IFN-β Expression in Human THP-1 Cells

**Figure 10:**
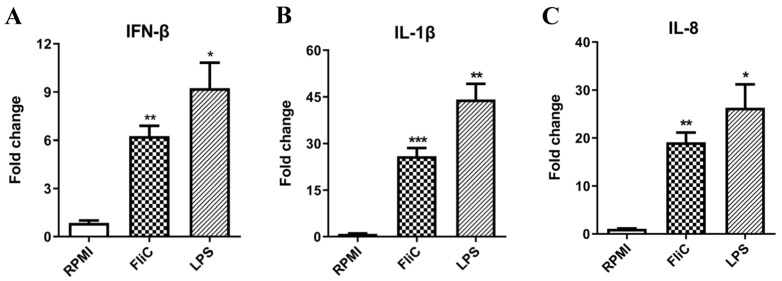
Flagellin stimulates IFN-β expression in human THP-1 cells. The THP-1 cells were stimulated with or without FliC (1 µg/mL) or LPS (0.1 µg/mL) for 5 h. The mRNA expression levels of cytokines IFN-β (**A**), IL-1β (**B**), and IL-8 (**C**) were analyzed by qRT-PCR. * *p* < 0.05, ** *p* < 0.01 and *** *p* < 0.001 compared with the control.

Human THP-1 cells were stimulated with FliC, or LPS as a positive control. After 5 h of stimulation, cells were harvested, and the mRNA expression levels of IFN-β, IL-1β, and IL-8 were detected by qRT-PCR. The results showed that *Salmonella* FliC could significantly upregulate the expression levels of IFN-β and the cytokines of IL-1β and IL-8 in human cells (Figure 10).

### 3.9. Flagellin Stimulates IFN-β Expression and Secretion in BMMs and BMDCs

**Figure 11:**
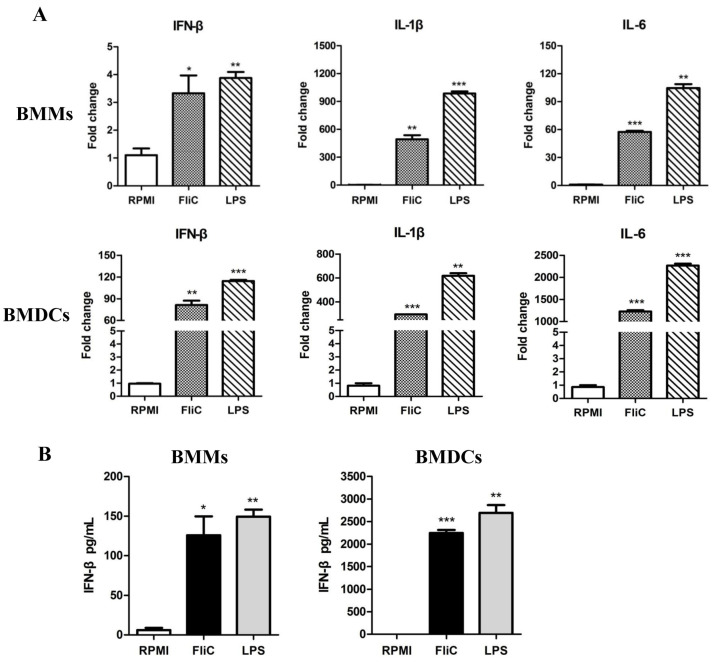
Flagellin stimulates IFN-β expression and secretion in BMMs and BMDCs. BMMs and BMDCs were isolated from C57BL/6 mice, and treated with or without FliC or LPS. The mRNA levels of inflammatory cytokines (IL-1β and IL-6) and IFN-β were analyzed by qRT-PCR, and the secretion levels of IFN-β in culture medium were measured using an ELISA set. (**A**) The mRNA levels of gene expression. (**B**) The protein level of IFN-β. * *p* < 0.05, ** *p* < 0.01, and *** *p* < 0.001 compared with the control.

The BMMs were stimulated with FliC, or LPS as a positive control. After 5 h, cells were harvested, RNA was extracted, and the mRNA levels of IFN-related genes were detected by qRT-PCR. After 12 h, the cell supernatant was collected and the secretion level of IFN-β was detected by ELISA. The results showed that FliC could significantly upregulate the expression of IFN-β and the known inflammatory cytokines (IL-1β and IL-6). Meanwhile, the protein level of IFN-β in the cell supernatant was consistent with the results of qRT-PCR (Figure 11). Furthermore, similar results were observed in another APC type, BMDCs, and gene upregulation was more significant in these cells than in BMMs (Figure 11), indicating that FliC significantly upregulated the secretion of IFN-β in BMMs and BMDCs ex vivo. 

### 3.10. Flagellin Promotes ISG Expression in BMMs and BMDCs

**Figure 12:**
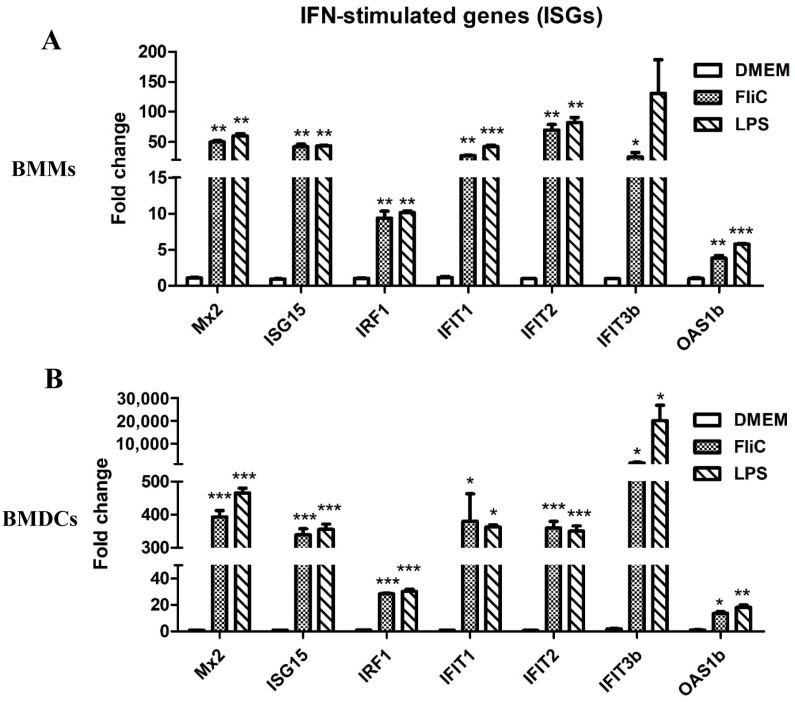
Flagellin promotes ISG expression in BMMs and BMDCs. BMMs and BMDCs were isolated from C57BL/6 mice, and treated with or without FliC or LPS. The mRNA levels of multiple ISGs in IFN-downstream signaling pathways were analyzed by qRT-PCR. (**A**) Detection of gene expression in BMMs. (**B**) Detection of gene expression in BMDCs. * *p* < 0.05, ** *p* < 0.01, and *** *p* < 0.001 compared with the control.

To further verify the effects of flagellin activation on IFN-β-related immune responses in APCs, multiple ISGs in IFN-related signaling pathways were detected by qRT-PCR. The results showed that FliC could significantly upregulate the expression of various ISGs (Mx2, ISG15, IFIT1, IFIT2, IFIT3b, and OAS1b) in both BMMs and BMDCs, which was consistent with the results in RAW264.7 cells. In addition, compared with BMMs, the expression levels of these genes were approximately 10 times higher in BMDCs (Figure 12), indicating that FliC has a stronger ability to activate immune responses in dendritic cells.

## 4. Discussion

Macrophages are an important component of the innate immune system [17]. They are activated during infection, inflammation, or injury, and promote the production of various host defense molecules [18,19]. These cytokines, including TNF, IL-1β, IL-6, and CXCL10, are essential to the function of macrophages. They mediate and connect innate immunity and acquired immunity [18,20]. The previous study showed that flagellin induces IFN-β production, and the IFN-β produced by the flagellin stimulation regulates anti-flagellin antibody class switching [21]. However, the regulatory network of flagellin activating immune responses was still unclear. In this study, we used an RNA-Seq approach to profile the differential gene expression in murine macrophage RAW264.7 cells in response to stimulation with FliC. As expected, FliC induced the expression of multiple genes involved in innate immune responses. These transcriptome data will help us to comprehensively understand the flagellin signaling network that activates specific innate immunity.

Kondo et al. confirmed that flagellin from *Bacillus subtilis* activated the NF-κB pathway in cultured A549 alveolar epithelial cells by microarray [22]. Clark et al. showed that airway epithelial cells cultured in monolayer and air-liquid interface have different transcriptional states following stimulation with flagellin isolated from *Pseudomonas aeruginosa* [23]. Im et al. found that bacterial flagellin stimulation elicited potent IL-17C expression in intestinal epithelial cells through a microarray approach [24]. Gao et al. used cDNA microarray to profile gene expression in *P. aeruginosa*–infected corneal epithelial cells with *P. aeruginosa* flagellin pretreatment [25]. In this study, RNA-Seq technology was used to assess the responses to *Salmonella* flagellin in macrophages. The RNA-Seq analysis revealed approximately 2204 DEGs when comparing the FliC-treated group with the control group, of which 1114 genes were upregulated. Moreover, most of the upregulated DEGs were enriched in the biological processes of immune regulation, immune defense, and immune responses, indicating that flagellin stimulation promotes the activation of macrophages and the production of innate immune responses. Innate immunity serves as an essential first-line defense against microbial pathogens and may also influence the subsequent adaptive immune response [26,27,28]. 

Cluster analysis of the DEGs revealed that immune response-related genes were significantly upregulated in response to FliC-induced macrophage activation. KEGG enrichment analysis of these genes showed that flagellin activated pathways, such as the TLR and NLR signaling pathways, and the NF-κB signaling pathway, which was consistent with previous reports [29,30]. TLR5 recognizes extracellular flagellin and subsequently recruits myeloid differentiation factor 88 (MyD88) to activate NF-κB [31], promoting the secretion of various inflammatory cytokines (such as IL-6, IL-12, and TNF-α) and chemokines [32]. Intracellular flagellin is recognized by the NLR family proteins NAIP5 and NAIP6 [33] and activates caspase-1. IL-1β precursors are cleaved and activated [34,35]. Therefore, several significantly upregulated genes involved in these pathways were verified by qRT-PCR. The inflammatory cytokines (IL-6, IL-1β, TNF-α, etc.) and chemokines (CXCL2, CCL2, CXCL10, CCL5, etc.) were confirmed to be significantly upregulated. The qRT-PCR and RNA-Seq results were closely matched, which was consistent with previous results from our laboratory [7,8] and those of others [36,37], including the report that recombinant *Legionella pneumophila* flagellin A (rflaA) induces RAW264.7 cells to secrete IL-6 and IL-1β in vitro [36]. Taken together, these findings demonstrate that flagellin stimulates macrophage activation. 

Immune recognition initiates innate immune responses leading to pathogen elimination, adaptive immunity stimulation, and trained immunity via cytokines and chemokines [38]. Previous studies showed that the rASP-1 adjuvant exerts its activities in vitro and in vivo through type I interferon signaling to promote DC maturation and T cell differentiation [39]. Although type I IFNs represent a family of cytokines with antiviral functions, type I IFNs have a critical role in Tfh differentiation, isotype class switching, and the induction of enhanced humoral immunity [40]. Type I interferon activates MHC class I-dressed CD11b^+^ conventional dendritic cells to promote protective antitumor CD8^+^ T cell immunity [41], therefore, functioning as a bridge between innate and adaptive immunity [42]. Interestingly, according to DEG enrichment analysis, many significantly upregulated DEGs were enriched in IFN-related signaling pathways. In particular, the expression of type I IFN (IFN-β) was significantly upregulated by flagellin stimulation of RAW264.7 cells, which was verified by qRT-PCR. Type I IFN binds to the interferon α/β receptor and induces the expression of more than 300 ISGs through the Janus kinase-signal transducer and activator of transcription (JAK-STAT) signaling pathway [43]. Our RNA-Seq analysis revealed that various ISGs were significantly upregulated in expression in FliC-treated RAW264.7 cells, including those encoding the myxovirus resistance proteins (Mx1 and Mx2), the IFIT family (IFIT1, IFIT2, and IFIT3b), and IRF3-dependent ISGs (ISG15, ISG20, OAS1b, etc). The mRNA levels of these ISGs were further confirmed by qRT-PCR. These ISGs are essential in limiting virus infection and transmission, as they can directly regulate key steps in innate and acquired immune responses [44]. Among them, mouse Mx1 was identified and cloned about 30 years ago as the first Mx protein family member [45]. Although IFIT family proteins are all induced by IFN, they control virus infection through distinct mechanisms [46]. In addition, the Trypsin-treated FliC group could not promote the up-regulated expression of IFN-β, indicating that flagellin itself had the ability to induce IFN-β.

Compared with macrophages, dendritic cells are well-characterized and potent APCs in the mammalian immune system. They play a crucial role in immune responses [47,48,49] and act as a link between the innate and adaptive immune systems. We further demonstrate that *Salmonella* FliC could upregulate IFN-β expression in mouse BMDCs and BMMs, as well as other inflammatory cytokines (IL-6 and IL-1β). Increased mRNA expression was accompanied by the secretion of IFN-β. This is an exciting finding because type I IFN plays an important role in both pathogen infection and immune system regulation [50]. In addition, the mRNA levels of upstream genes encoding IRFs and downstream ISGs (Mx2, ISG15, IFIT1, IFIT2, and OAS1b) were significantly increased in BMDCs and BMMs, which confirmed that flagellin was involved in IFN-related signaling pathways. The protein products encoded by these ISGs work alone or in concert to achieve multiple cellular outcomes, including antiviral defense, antiproliferative activities, and stimulation of adaptive immunity [51]. Thus, flagellin may exert its adjuvant activity by inducing ISGs expression beyond the activation of TLR5 and NLRC4.

As a pleiotropic cytokine, type I IFN has been widely recognized for its antiviral effects [44]. Through RNA-Seq, we found that in flagellin-stimulated macrophages, in addition to the upregulation of the TLR and NLR signaling pathways, genes related to the RIG-I receptor (RLR) signaling pathways were also significantly upregulated. RLRs can also activate the production of downstream type I IFN through mitochondrial antiviral signaling protein [52]. This finding suggested that either the TLR or RLR signaling pathway may be the key pathway to regulate FliC-induced IFN-β-related signaling of RAW264.7 cells. Therefore, the specific signaling pathway of flagellin to activate IFN-β is worthy of further study. These findings provide a basis for further studies into the adjuvant mechanism of the action of FliC.

## 5. Conclusions

In summary, the comprehensive transcript patterns of RAW264.7 cells stimulated by *Salmonella* flagellin were analyzed by RNA-Seq, and it was found that immune-related cytokines or chemokines showed a strong response to FliC. Importantly, FliC significantly increased the expression of IFN-β and ISGs in RAW264.7 macrophages, which was confirmed by qRT-PCR. Moreover, flagellin activation of IFN-β-related immune responses was also demonstrated in BMMs and BMDCs. Taken together, these results suggested that *Salmonella* flagellin activated a complex network of innate immunity, providing new insight into the immune mechanisms of flagellin adjuvant.

## Figures and Tables

**Table 1:** Sequences of primers used for the quantitative real-time PCR.

Gene	Forward Primer (5′-3′)	Reverse Primer (5′-3′)	Product Size (bp)	Accession No.
IL-1β	gcccatcctctgtgactcat	aggccacaggtattttgtcg	230	NM_008361.4
IL-18	gacagcctgtgttcgaggat	tggatccatttcctcaaagg	188	NM_008360.2
IL-6	agttgccttcttgggactga	tccacgatttcccagagaac	159	NM_031168.2
TNF-α	agcccccagtctgtatcctt	ctccctttgcagaactcagg	212	NM_013693.3
CCL2	aggtccctgtcatgcttctg	tctggacccattccttcttg	249	NM_011333.3
CCL3	agattccacgccaattcatc	ctcaagcccctgctctacac	223	NM_011337.2
CCL4	cccacttcctgctgtttctc	gaggaggcctctcctgaagt	238	NM_013652.2
CCL5	tgccaacccagagaagaagt	agatgcccattttcccagga	184	NM_013653.3
CXCL2	aagtttgccttgaccctgaa	aggcacatcaggtacgatcc	180	NM_009140.2
CXCL10	ggatggctgtcctagctctg	ataaccccttgggaagatgg	208	NM_008176.3
IFN-β	ccctatggagatgacggaga	ctgtctgctggtggagttca	161	NM_010510.1
IRF1	gcaaaaccaagaggaagctg	cagagagactgctgctgacg	186	NM_008390.2
IRF9	gtctggaagactcgcctacg	tggtcctcccattttccata	220	NM_001159417.1
Mx1	tctgtgcaggcactatgagg	gcctctccactcctctcctt	247	NM_010846.1
Mx2	cccagaggcagtggtattgt	acatttggggagctgacatc	227	NM_013606.1
ISG15	aagaagcagattgcccagaa	tctgcgtcagaaagacctca	217	NM_015783.3
ISG20	ccatggactgtgagatggtg	agcttgcctttcagaagctg	229	NM_020583.5
IFIT1	aggctggagtgtgctgagat	tctggatttaaccggacagc	224	NM_008331.3
IFIT2	caccttcggtatggcaactt	gcaaggcctcagaatcagac	181	NM_008332.3
IFIT3b	cgagcaaaaatgtgctttga	gctccccttcagcttcttct	190	NM_001005858.3
OAS1b	accgtcttggaactggtcac	atgttccttgttgggtcagc	155	NM_001083925.1
GAPDH	aactttggcattgtggaagg	acacattgggggtaggaaca	223	NM_001289726.1
hIFN-β	cattacctgaaggccaagga	cagcatctgctggttgaaga	178	NM_002176.4
hIL-1β	gggcctcaaggaaaagaatc	ttctgcttgagaggtgctga	205	NM_000576.3
hIL-8	gtgcagttttgccaaggagt	ctctgcacccagttttcctt	196	BC013615.1
hGAPDH	gagtcaacggatttggtcgt	ttgattttggagggatctcg	238	NM_002046.7

## Data Availability

The RNA-Seq datasets used in this study have been deposited in the NCBI Gene Expression Omnibus under the accession number GSE200796.

## Supplementary Materials

The following supporting information can be downloaded at: https://www.mdpi.com/article/10.3390/cimb45040183/s1, Table S1: The top upregulated genes (log2-fold ≥ 2.0) upon *Salmonella* FliC stimulation; Table S2: The top downregulated genes (log2-fold ≤ −1.0) upon *Salmonella* FliC stimulation.
